# KIR/HLA genotype combinations are determinants of Natural Killer (NK) cell mediated antibody-dependent cellular cytotoxicity (ADCC) potency

**DOI:** 10.1186/1742-4690-9-S2-P187

**Published:** 2012-09-13

**Authors:** MS Parsons, P Zanoni, B Tallon, S Miconiatis, N Shoukry, J Bruneau, CM Tsoukas, NF Bernard

**Affiliations:** 1 Research Institute McGill University Health Center, Montreal, Canada; 2 Centre de Recherche du Centre Hospitalier de l'Université de Montréal, Canada

## Background

Several allelic variants of the killer immunoglobulin-like receptor 3DL1 (KIR3DL1) in combination with their HLA-Bw4 ligands, are associated with protection from HIV infection and/or time to AIDS. The HLA-B*57+KIR3DL1*h/*y genotype combination (KIR3DL1*h/*y encodes high expression receptors) has the most potent effect on slowing time to AIDS in HIV infected individuals. Although this HLA/KIR combination confers natural killer (NK) cells with high functional potential, the mechanism of the AIDS protective effect remains unknown. Indeed, NK cells expressing 3DL1 fail to degranulate upon exposure to autologous HIV-infected CD4+ T-lymphocytes. As HLA-Bw4/KIR3DL1 combinations also endow NK cells with antibody-dependent cellular cytotoxicity (ADCC) functional potential, we hypothesized that NK cells from individuals carrying HLA-B*57+3DL1*h/*y mediate more potent ADCC than NK cells from individuals with less protective HLA/KIR combinations.

## Methods

We compared the ability of NK cells from 39 HIV-uninfected individuals carrying HLA-B*57+3DL1*h/*y (n=11), other HLA-Bw4+3DL1 combinations (n=18), or 3DL1 in the absence of HLA-Bw4 (n=10) to mediate anti-HIV ADCC. Anti-HIV ADCC was measured as the ability of NK cells to deliver granzyme B to HIV gp120-coated CEM.NKr.CCR5 cells (i.e. percent of granzyme B positive cells) in the presence of a common source of anti-HIV antibodies. This GranToxiLux assay efficiently measures ADCC, as target cells do not receive granzyme B in the presence of antibodies from seronegative individuals or F(ab')2 fragments of anti-HIV antibodies.

## Results

NK cells from HLA-B*57+3DL1*h/*y carriers mediate more potent ADCC (13.4+/-2.61%) than those from individuals carrying other HLA-Bw4+3DL1 combinations (5.15+/-1.54%) or 3DL1 in the absence of HLA-Bw4 (2.78+/-1.48%) (p=0.002, Kruskal-Wallis test, p<0.05 and p<0.01 respectively, Dunn’s Multiple Comparisons test).

## Conclusion

Therefore, HLA/KIR dependent NK cell “education” is a determinant of ADCC functional potential, and should be taken into consideration when evaluating ADCC as a correlate of disease progression or vaccine efficacy.

